# Supplementary figures and images for: Using lifestyle information in polygenic modeling of blood pressure traits: a simple method to reduce bias
Source: bioRxiv. 2024 Jun 8:2024.06.05.597631. Preprint. [Version 1] doi: 10.1101/2024.06.05.597631 (PMC11185601; doi:10.1101/2024.06.05.597631)

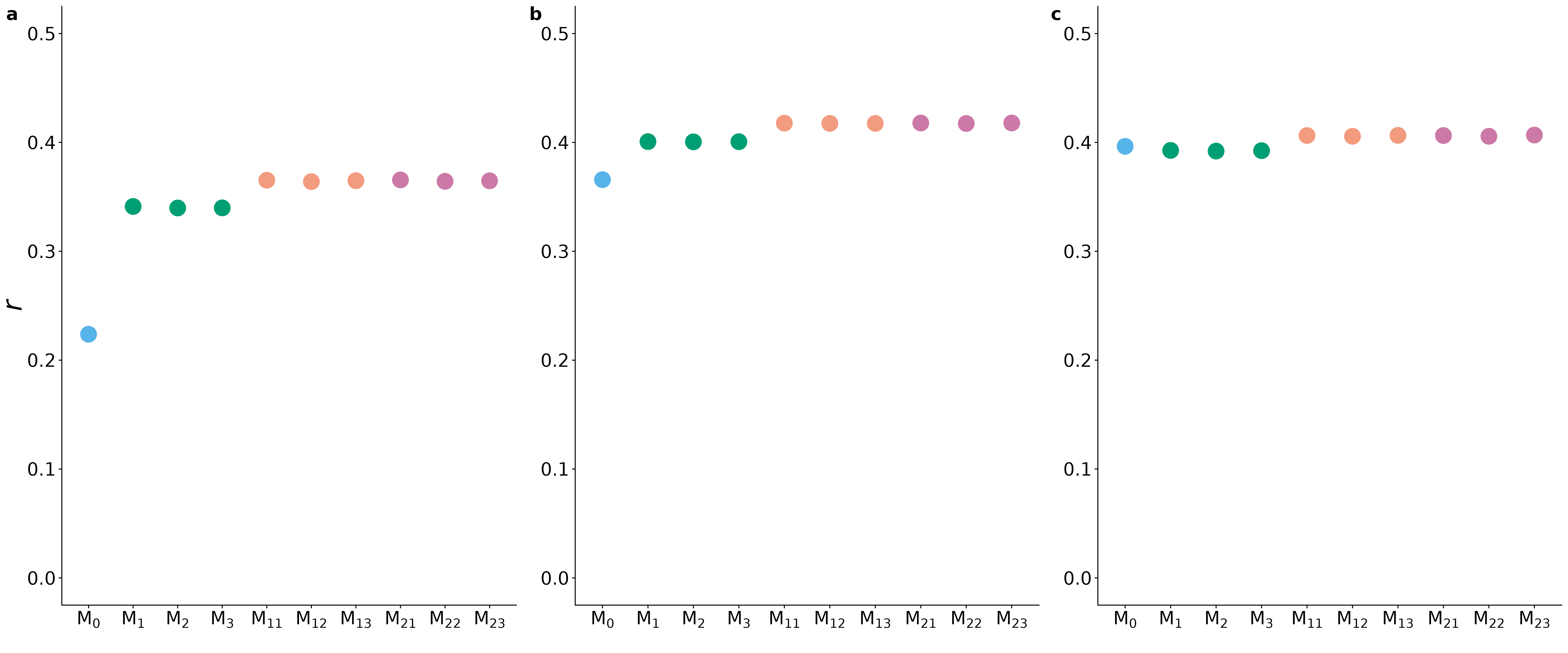

Supplement: Supplement 4 — S1 Fig. Accuracy of prediction measured as the correlation coefficient (r) between true phenotypes and predicted phenotype within cohorts for a) diastolic pressure, b) systolic pressure and c) pulse pressure. The colors indicate the broad class of models, i.e., including genetic effects only (light blue), lifestyle effects only (green), genetic and lifestyle effects (orange), and genetic, lifestyle and interaction effects (purple). [file media-4.jpg]

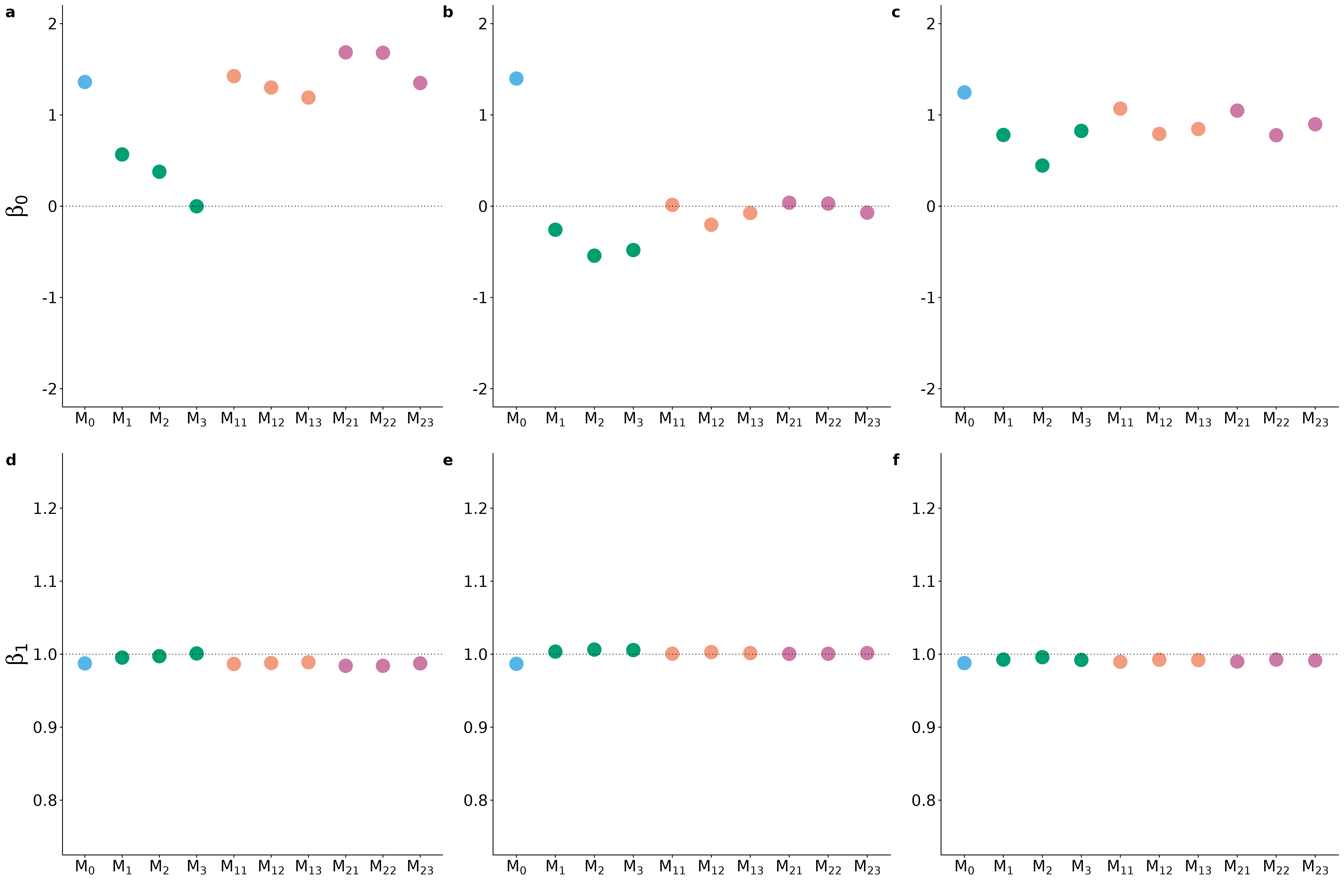

Supplement: Supplement 5 — S2 Fig. Bias of predictions measured as the intercept [β0; panels a), b) and c) for diastolic pressure, systolic pressure, and pulse pressure, respectively] and slope [β1; panels d), e) and f) for diastolic pressure, systolic pressure, and pulse pressure, respectively] of the regression of the true phenotypes on the predicted phenotypes within cohorts. The colors indicate the broad class of models, i.e., including genetic effects only (light blue), lifestyle effects only (green), genetic and lifestyle effects (orange), and genetic, lifestyle and interaction effects (purple). The dotted lines indicate the expected values of β0 and β1 for unbiased predictions. [file media-5.jpg]
